# Exploring the influence of slaughterhouse type and slaughtering steps on *Campylobacter jejuni* contamination in chicken meat: A cluster analysis approach

**DOI:** 10.1016/j.heliyon.2024.e32345

**Published:** 2024-06-04

**Authors:** Chalita Jainonthee, Warangkhana Chaisowwong, Phakamas Ngamsanga, Tongkorn Meeyam, Fernando Sampedro, Scott J. Wells, Duangporn Pichpol

**Affiliations:** a Veterinary Public Health and Food Safety Centre for Asia Pacific (VPHCAP), Faculty of Veterinary Medicine, Chiang Mai University, Chiang Mai, 50100, Thailand; b Research Center for Veterinary Biosciences and Veterinary Public Health, Faculty of Veterinary Medicine, Chiang Mai University, Chiang Mai, 50100, Thailand; c Department of Veterinary Biosciences and Veterinary Public Health, Faculty of Veterinary Medicine, Chiang Mai University, Chiang Mai, 50100, Thailand; d Environmental Health Sciences Division, School of Public Health, University of Minnesota, Minneapolis, MN, 55455, USA; e Department of Veterinary Population Medicine, College of Veterinary Medicine, University of Minnesota, St. Paul, MN, 55108, USA

**Keywords:** Backyard, Broiler, *Campylobacter jejuni*, Cluster analysis, Enumeration, K-modes clustering, Slaughterhouse

## Abstract

*Campylobacter jejuni* (*C. jejuni*), a foodborne pathogen, poses notable hazards to human health and has significant economic implications for poultry production. This study aimed to assess *C. jejuni* contamination levels in chicken carcasses from both backyard and commercial slaughterhouses in Chiang Mai province, Thailand. It also sought to examine the effects of different slaughtering practices on contamination levels and to offer evidence-based recommendations for reducing *C. jejuni* contamination. Through the sampling of 105 chicken carcasses and subsequent enumeration of *C. jejuni*, the study captured the impact of various slaughtering practices. Utilizing k-modes clustering on the observational and bacterial count data, the research identified distinct patterns of contamination, revealing higher levels in backyard operations compared to commercial ones. The application of k-modes clustering highlighted the impact of critical slaughtering practices, particularly chilling, on contamination levels. Notably, samples with the lowest bacterial counts were typically from the chilling step, a practice predominantly found in commercial facilities. This observation underpins the recommendation for backyard slaughterhouses to incorporate ice in their post-evisceration soaking process. Mimicking commercial practices, this chilling method aims to inhibit *C. jejuni* growth by reducing carcass temperature, thereby enhancing food safety. Furthermore, the study suggests backyard operations adopt additional measures observed in commercial settings, like segregating equipment for each slaughtering step and implementing regular cleaning protocols. These strategic interventions are pivotal in reducing contamination risks, advancing microbiological safety in poultry processing, and aligning with global food safety enhancement efforts.

## 1 Introduction

Foodborne diseases pose a significant threat to global public health, with bacterial infections being of particular concern. *Campylobacter jejuni*, one of these bacterial pathogens, is a leading cause of gastroenteritis worldwide [[1], [2], [3]]. *C. jejuni* has been identified as a significant reservoir in poultry [4,5], which is a staple protein source in many cultures. As a crucial link in the food manufacturing and supply chain, slaughterhouses could substantially influence the spread of these pathogens. It is evident that *C. jejuni* infection mostly occurs on farms, while contamination of poultry carcasses occurs during primary processing [6,7]. Therefore, the procedures and practices at these facilities may have implications for pathogen control, food safety, and public health. This context is enriched by considering the variation between various types of slaughterhouses, such as small-scale backyard operations versus larger, industrial-scale commercial facilities. Understanding these dynamics could facilitate the identification of key determinants of contamination and pave the way for the development of more effective food safety interventions.

Previous research has unquestionably improved our comprehension of *C. jejuni* infection in poultry and contamination in chicken carcasses by clarifying its biology, pathogenicity, and transmission routes [[8], [9], [10]]. However, our understanding of the influence of slaughtering practices on *C. jejuni* contamination in various categories of slaughterhouses remains limited. Although there is a wide range of research examining the prevalence of the bacteria at various phases of the production chain [[11], [12], [13], [14], [15]] and the impact of slaughtering practices on the contamination of poultry meat with *Campylobacter* [[16], [17], [18]], there is still a need for a more comprehensive investigation into the specific contributions of different types of slaughterhouses and individual steps in the slaughtering process to the contamination of chicken meat with *C. jejuni*. In addition, the existing literature tends to neglect backyard slaughterhouses, where biosecurity measures may vary and differ significantly from commercial operations. This gap in knowledge has prevented a holistic approach to the problem, hampering the design of comprehensive and effective control strategies. The present study is designed to fill this gap by comparing contamination levels in chicken carcasses from backyard and commercial slaughterhouses, considering their slaughtering practices, and examining the impact of these practices on *C. jejuni* contamination. The results are anticipated to improve our comprehension of the differential routes of contamination in various settings, thereby informing more targeted interventions.

As one of the world's leading exporters of poultry meat, Thailand plays a vital role in the international food supply chain. The country's poultry industry consists of a complex mosaic of commercial and backyard slaughterhouses, each of which caters to distinct market demands. Commercial slaughterhouses process poultry meat primarily for high-end markets, such as supermarkets and international export, thereby establishing stringent hygiene and food safety standards. However, Thailand's local consumption includes both commercial and domestic slaughterhouse products. Typically, smaller and less regulated, backyard operations serve local markets and rural communities. This diverse landscape of poultry production and slaughtering practices in Thailand presents unique challenges in controlling *C. jejuni* contamination, making it a compelling context for this study.

To address the problem of *C. jejuni* contamination in chicken carcasses, an innovative analytical strategy that goes beyond descriptive and inferential statistics is necessary. This study applies k-modes clustering [19], a potent machine learning technique, to this intricate problem. Typically utilized in data mining, k-modes clustering allows us to classify observations into clusters based on patterns of categorical variables [20]. In the context of our study, this methodology permits us to categorize various slaughtering practices according to their similarities and analyze how these groupings correspond to *C. jejuni* contamination levels. By doing so, we can identify distinct contamination patterns across various slaughtering practices and between different types of slaughterhouses. This nuanced, data-driven strategy is anticipated to yield insights that could result in more precise and efficient measures to mitigate *C. jejuni* contamination.

The primary objective of this study was to compare and contrast the operational procedures and practices of backyard slaughterhouses and commercial slaughterhouses in Chiang Mai province, Thailand, and to evaluate their impact on *C. jejuni* contamination levels in chicken carcasses. Using k-modes clustering, this study aimed to determine the relationship between specific slaughtering steps, slaughterhouse types, and *C. jejuni* counts in chicken carcasses. By revealing potential contributory factors to contamination at various slaughtering steps and across different types of slaughterhouses, this study aims to provide insights that can enhance contamination control measures and hygiene practices, ultimately improving public health and poultry industry standards.

## 2 Materials and methods

### 2.1 Study area and categorization of slaughterhouses

Chiang Mai province, the highest poultry production in the upper northern region of Thailand, was chosen as the study area. In order to collect carcass samples, the study defined two categories of slaughterhouses: backyard slaughterhouses and commercial slaughterhouses. Depending on consumer demand, a backyard slaughterhouse provides a small quantity of chicken slaughtering capacity (less than 50 birds per day). The vendors typically use their home or a nearby home to operate the slaughtering process. The majority of the chickens supplied to this form of slaughterhouse comes from small farms and backyard rearing, and distribution of meat products was concentrated in rural fresh markets. A commercial slaughterhouse is a facility that has the capacity to slaughter more than 5000 birds per day. This variety of slaughterhouse has made substantial infrastructure and equipment investments. The majority of broilers comes from farms owned by commercial broiler companies or contract farms with moderate to high biosecurity. Commercial chicken meat products are distributed to urban fresh markets, supermarkets, restaurants, hotels, and company retail stores in Chiang Mai and other provinces in the upper northern region of Thailand. The slaughterhouses were selected using a convenience sampling of all eligible slaughterhouses, with each slaughterhouse agreeing to participate in the study voluntarily. The selection of backyard slaughterhouses was contingent on receiving approval from the proprietors, while the selection of commercial slaughterhouses was contingent on receiving approval from the managers.

### 2.2 Slaughterhouse profiles

Based on inquiries, the total slaughtering capacity of commercial slaughterhouses was 31,000 birds per day. Approximately 8370 chicken carcasses, accounting for 27 % of the daily commercial production, were transported to Chiang Mai on a daily basis. The remaining carcasses were distributed to various provinces in the upper northern region of Thailand, including Chiang Rai, Nan, Phayao, Phrae, Mae Hong Son, Lampang, and Lamphun. Based on data collected from four backyard slaughterhouses, it was determined that the average daily slaughtering capacity of these facilities was 18 birds. Additionally, experts from the Department of Livestock Development (DLD) estimated that there were approximately 60 backyard slaughterhouses in Chiang Mai. This estimation was made due to the non-compliance of most backyard facilities with the DLD's slaughtering registration regulations. The estimated daily output of backyard chicken carcasses was 1080 birds per day.

**Fig. 1:**
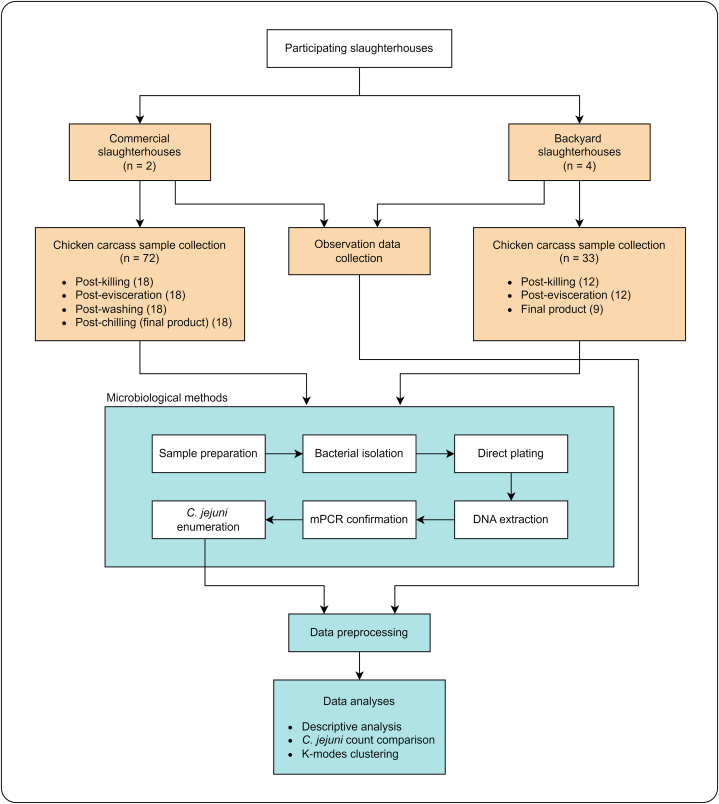
Workflow diagram of the study, showing the inclusion of slaughterhouses through convenience sampling and outlining subsequent research steps, from the initial collection of chicken carcass samples and data gathering to microbiological methods and data analyses. For details on the specific points where samples were collected at each step in each slaughterhouse, refer to Fig. 2.

**Fig. 2:**
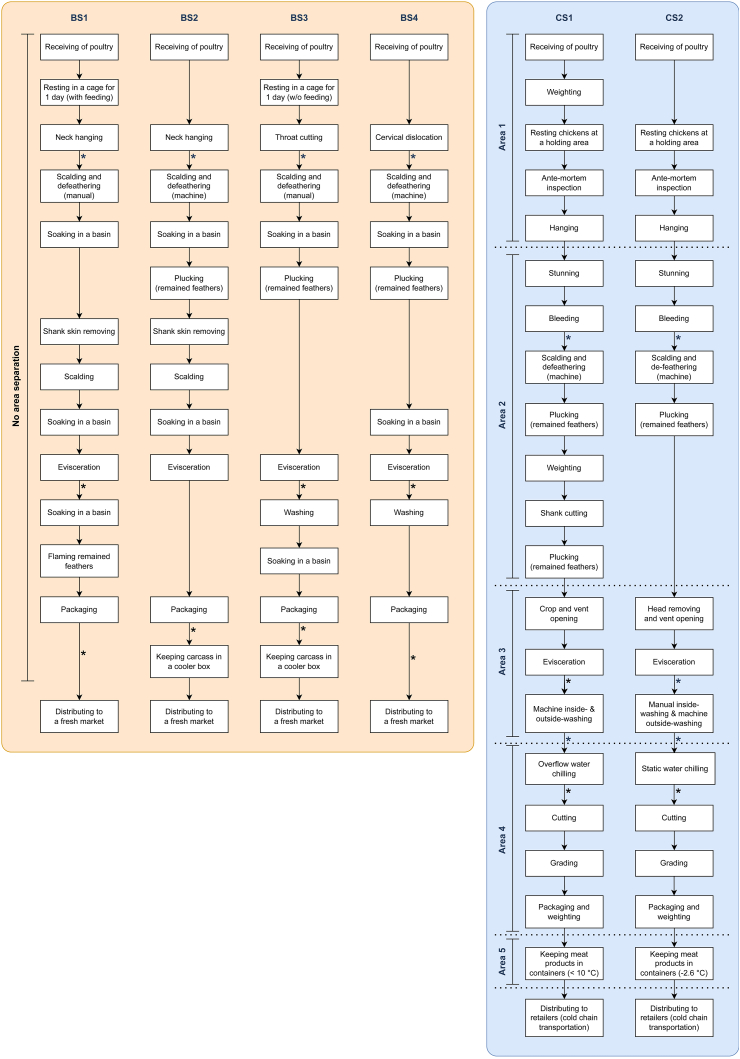
Chicken slaughter process of each slaughter facility. BS represents a backyard slaughterhouse and CS represents a commercial slaughterhouse. The asterisks (*) indicate steps where carcass samples were collected.

This investigation included two commercial slaughterhouses and four backyard slaughterhouses. The workflow of the study, illustrating the process of engaging slaughterhouses through convenience sampling and outlining the subsequent research steps, is depicted in Fig. 1. Additionally, Fig. 2 illustrates the slaughtering process at each slaughterhouse. Observations and inquiries were made to gather more information about slaughtering practices and slaughterhouse operations.

### 2.3 Sample collection

From June to October 2016, a total of 105 carcass samples were collected from backyard and commercial slaughterhouses. Each commercial slaughterhouse was sampled three times, whereas each backyard slaughterhouse was sampled only once. Using a random sampling technique, three samples per step were selected at random within each slaughterhouse. Thirty-three carcasses were randomly collected from four backyard slaughterhouses along the slaughter line at three critical steps: post-killing, post-evisceration, and before distribution to markets. In one backyard plant, three samples were excluded from the sampling plan because evisceration was the final step of operation. The remaining samples (*n* = 72) were collected from two commercial slaughterhouses along the slaughter line at four critical steps: post-killing, post-evisceration, post-washing, and post-chilling (presumed final product prior to distribution). In this investigation, external fecal contamination during transportation was not evaluated. Separately, a carcass sample was collected in a sterile plastic container. After collection, carcass samples were stored at temperatures below 4 °C in ice cases and transported within 3 h to the Central Laboratory of the Faculty of Veterinary Medicine, Chiang Mai University.

### 2.4 Microbiological methods

#### 2.4.1 Sample preparation

A total of 10 g of cecal content was extracted aseptically from intact ceca of carcasses collected post-killing, following laboratory transport. Then, 10 g of cecal content was combined with 90 ml of phosphate buffered saline (PBS) and homogenized by a stomacher for 2 min. Using a stomacher, 10 ml of the diluent was combined with 90 ml of Bolton selective enrichment broth (Oxoid, UK) containing 5 % (v/v) laked horse blood (Thermo Scientific, UK) for 2 min. The initial diluent preparation was 10^−1^, which was then serially diluted 10-fold using PBS to yield 10^−2^ and 10^−3^ dilutions. Three dilutions of the sample were prepared to detect and count *C. jejuni* using the direct plating method.

In order to prepare the rinse solution, the rinse method (ISO 17604:2003) [21] was applied to a chicken carcass collected after evisceration, washing, and chilling (final product) in a sterile plastic bag and manually shaken in 200 ml PBS for 5 min. The rinse solution was homogenized for 2 min using a stomacher, then poured into a sterile container. Then, 25 ml of the solution was combined with 225 ml of Bolton selective enrichment broth containing 5 % (v/v) laked horse blood and homogenized by a stomacher for 2 min. The initial formulation of the solution was 10^−1^, which was then serially diluted 10-fold with PBS to yield dilutions of 10^−2^ and 10^−3^. Three dilutions of the sample were prepared for *C. jejuni* detection and enumeration using the direct plating method.

#### 2.4.2 Bacterial isolation

Detection of *C. jejuni* was modified from ISO 10272–1:2006 [22] by using multiplex PCR (mPCR) instead of biochemical assays for species confirmation. The initial solution in a sterile container was incubated at 37 °C for 6 h and 41.5 °C for 42 h, respectively, in a microaerobic atmosphere containing 5 % O_2_, 10 % CO_2_, and 85 % N_2_ generated with a CampyGen™ 2.5L (Oxoid, UK). At 41.5 °C for 42 h, a single loopful of culture was streaked on selectively modified charcoal cefoperazone deoxycholate agar (mCCDA; Oxoid, UK). Then, five presumptive *Campylobacter* colonies were streaked on non-selective Columbia blood agar (CBA; Oxoid, UK) supplemented with 5 % (v/v) sterile defibrinated sheep blood (Clinag, Thailand) and incubated at 41.5 °C for 42 h under microaerobic conditions. After incubation, colonies on CBA were preserved for DNA extraction in 1 ml of PBS. Minimum of five per sample were confirmed by mPCR.

#### 2.4.3 Direct plating technique

From the chicken carcass rinse, duplicate 10-fold serial dilutions ranging from 10^−1^ to 10^−3^ were prepared, and 100 μl of each dilution was spread in duplicate on mCCDA and incubated at 41.5 °C for 42 h under microaerobic conditions. Prior to use, the agar plates were dried for 30 min to avoid swarming of the colonies. After incubation, all colonies with a metallic, flat, and moist grayish appearance were enumerated, and the number of colonies at each dilution was recorded. Five presumed *Campylobacter* colonies of each dilution were then separately streaked onto CBA supplemented with 5 % (v/v) sterile defibrinated sheep blood and incubated at 41.5 °C for 42 h under microaerobic conditions. Following incubation, colonies on CBA were screened with oxidase and catalase tests, swabbed, and preserved in 1 ml of PBS for DNA extraction. Minimum of five per sample were confirmed by mPCR.

#### 2.4.4 DNA extraction and mPCR confirmation

Pure culture preserved in 1 ml PBS was centrifuged at 10,000 rpm for 5 min to extract DNA using the boiling method, and the supernatant was removed. The pellet was resuspended in 200 μl of distilled water. The sample was centrifuged for 5 min at 10,000 rpm, and the supernatant was removed. The pellet was resuspended in 500 μl of distilled water. The bacterial suspension was boiled for 10 min at 100 °C in a heat block and then chilled for 10 min at 0 °C in an icebox. The sample was then centrifuged at 14,000 rpm for 1 min. Before use, the supernatant was transferred to a new microcentrifuge tube and stored at −20 °C.

Identification of *16S rRNA, mapA* and *ceuE* genes were performed using a mPCR assay utilizing primers previously published by Denis et al. (1999) [23]. The primers were designed for identification and differentiation of *C. jejuni* and *C. coli*. The forward primer for *Campylobacter* genus (targeted *16S rRNA* gene) was 5′-ATCTAATGGCTTAACCATTAAAC-3’ (CCCJ609-F), the reverse primer was 5′-GGACGGTAACTAGTTTAGTATT-3’ (CCCJ609-R). The forward primer for *jejuni* species (targeted *mapA* gene) was 5′-CTATTTTATTTTTGAGTGCTTGTG-3’ (mapA-F), the reverse primer was 5′-GCTTTATTTGCCATTTGTTTTATTA-3’ (mapA-R). The forward primer for *coli* species (targeted *ceuE* gene) was 5′-AATTGAAAATTGCTCCAACTATG-3’ (ceuE-F), the reverse primer was 5′-TGATTTTATTATTTGTAGCAGCG-3’ (ceuE-R). The 15-μl PCR reaction mixtures contained 1.5 μl DNA template, 7.5 μl Quick Taq™ HS DyeMix PCR reagent (Toyobo, Japan), 0.11 μM CCCJ609-F and CCCJ609-R primers, 0.42 μM mapA-F, mapA-R, ceuE-F and ceuE-R primers. The final volume was adjusted to 15 μl. The amplification reactions were performed on a thermocycler with the following program: an initial denaturation step at 95 °C for 10 min, amplification consisting of 95 °C for 30 s, 59 °C for 1.5 min, 72 °C for 1 min, then repeat amplification to 35 cycles followed by final extension at 72 °C for 10 min. The temperature was held at 4 °C. PCR products were analyzed by 1.5 % agarose gel electrophoresis for 30 min at 110V. Regarding the primer pairs used for mPCR experiments, the product sizes were 857 bp, 589 bp, 462 bp for *Campylobacter* genus, *jejuni*, and *coli* species, respectively. Only samples positive for *C. jejuni* were included in data analysis.

#### 2.4.5 *C. jejuni* enumeration

Positive DNA samples for *C. jejuni* were identified by the presence of *16s rRNA* and *mapA* genes. The quantity of *C. jejuni* was determined by tracing to the recorded dilution and number of typical colonies. The colony count of each sample was determined using ISO 10272–2:2006 [24]. Counts of *C. jejuni* were log-transformed prior to data analysis. Calculated bacterial counts for the samples were expressed as log CFU/unit (log CFU/g for the cecal sample and log CFU/ml for the carcass rinse sample). The detection limit for enumeration was 100 CFU/g for cecal samples and 10 CFU/ml for carcass rinse samples [18,25]. Half of the enumeration limit was designated as counts for samples that were below the enumeration limit or tested negative [26]. For the backyard slaughterhouse where evisceration was the final step, the counts from samples collected at that step were repetitive used as the final products for data analysis.

### 2.5 Data Preprocessing

*C. jejuni* counts and additional observational variables representing the sample and the slaughtering practices were input into a spreadsheet before analyzing. Observational data collected during a slaughterhouse visit were recorded as binary inputs, including the practice of resting chickens before killing (0 = Slaughter immediately after transport to a slaughterhouse, 1 = Rest for at least 30 min before killing), feeding (0 = No feeding, 1 = Feeding chickens before killing), soaking (a practice of gathering chicken carcasses into a water container before the step of sample collecting; 0 = No, 1 = Yes), washing (a practice of individual carcass washing, manually or by a washing machine before the step of sample collecting; 0 = No, 1 = Yes), chilling (a cooling process to reduce the temperature of the chicken carcass by applying chilled water to the whole carcass; 0 = No, 1 = Yes), water source (1 = Tap water, 2 = Ground chlorine-treated water). Additionally, the ambient temperature or average carcass temperature (if applicable) was recorded prior to sample collection and input as a variable representing the temperature before a step of sample collection.

After exploring the influence of each variable, soaking was removed since all carcasses collected from post-evisceration to final products were processed using a different type of soaking, namely scalding or carcass chilling. In addition, water source was also removed from the analysis due to insignificant influence on counts and unknown concentration of chlorine treated for tap water. Value input was considered from only the variables involving the carcass before it was collected, e.g. for the samples collected at post-evisceration, only the variables of ‘count’, ‘rest’, ‘feed’, and ‘soak’ were input depended on actual practices in the slaughterhouse, while the other variables were input as zero. Thus, for cecal samples, the variables of ‘count’, ‘rest’, and ‘feed’ were considered for the analysis while ‘count’, ‘rest’, ‘feed’, ‘wash’, ‘chill’, and ‘temperature’ were considered for carcass rinse samples.

The data was preprocessed to prepare for k-modes clustering [19], which was used for clustering categorical variables. Specifically, the continuous variable ‘count’ and ‘temperature’ were transformed into categorical variables ‘count_cat’ and ‘temp_cat*’*, respectively. For ‘count’, it was divided into three categories: ‘Low’, ‘Medium’, and ‘High’, based on its quantile distribution. The division was done using the *numpy. quantile* and *pandas. cut* functions in Python, which created bins based on the 33rd and 67th percentiles of the count distribution. Similarly, ‘temperature’ was divided into five categories: ‘Very Low’, ‘Low’, ‘Medium’, ‘High’, and ‘Very High’, based on its quantile distribution. The division for ‘temperature’ created bins based on the 20th, 40th, 60th, and 80th percentiles of the temperature distribution.

### 2.6 Data analyses

A descriptive analysis was performed to provide a profile of contamination for each slaughterhouse, including mean counts for each processing step and differences in counts between steps. On the basis of sample type and sample preparation methods, groups of cecal and carcass rinse samples were separated for analysis as previously described in Data Preprocessing section.

The recorded *C. jejuni* count data were first examined for normality using the Shapiro-Wilk test, as this determined the appropriate statistical tests for comparison. This was done in order to provide a general comparison of the samples taken from commercial and backyard plants. When comparing two groups, if the data from both groups were normally distributed and homoscedastic, an independent samples *t*-test was used. If the data were not normally distributed or the groups had unequal variances by performing a Levene's test, a non-parametric alternative, the Mann-Whitney *U* test, was used instead. In the study, four comparisons were made as follows between backyard and commercial slaughterhouses: (1) the count at the post-killing step; (2) the count at the post-evisceration step; (3) the count at the final product step; and (4) the total rinse sample counts (combining post-evisceration, post-washing, and final product). All analyses were performed in Python, using the ‘SciPy’ package for statistical testing. The level of significance for all statistical tests was set at 0.05.

In order to determine the influence of practices on the contamination of *C. jejuni* in chicken carcasses, samples from the same preparation method were combined into the same dataset for analyses, regardless of slaughterhouse type. The k-modes clustering algorithm was applied on the dataset to identify distinct clusters within the data. The number of clusters was set to three with an aim to find differences between clusters of low, medium, and high counts. The clustering algorithm was initialized using the ‘Huang’ method, which is suitable for categorical data. The algorithm was run with 10 different centroid initializations (n_init parameter set to 10) and the best clustering result in terms of the cost function was chosen. To better understand the characteristics of the clusters, the distribution of each feature within each cluster was visualized using bar plots. All data analyses were performed in Python. The ‘pandas’ library was used for data manipulation. The ‘numpy’ was used for numerical computations and creating bins for categorizing continuous variables. The k-modes clustering was conducted using the ‘kmodes’ library. Additionally, ‘matplotlib’ and ‘seaborn’ were used for data visualization.

## 3 Results

### 3.1 Slaughtering practices

**Fig. 3:**
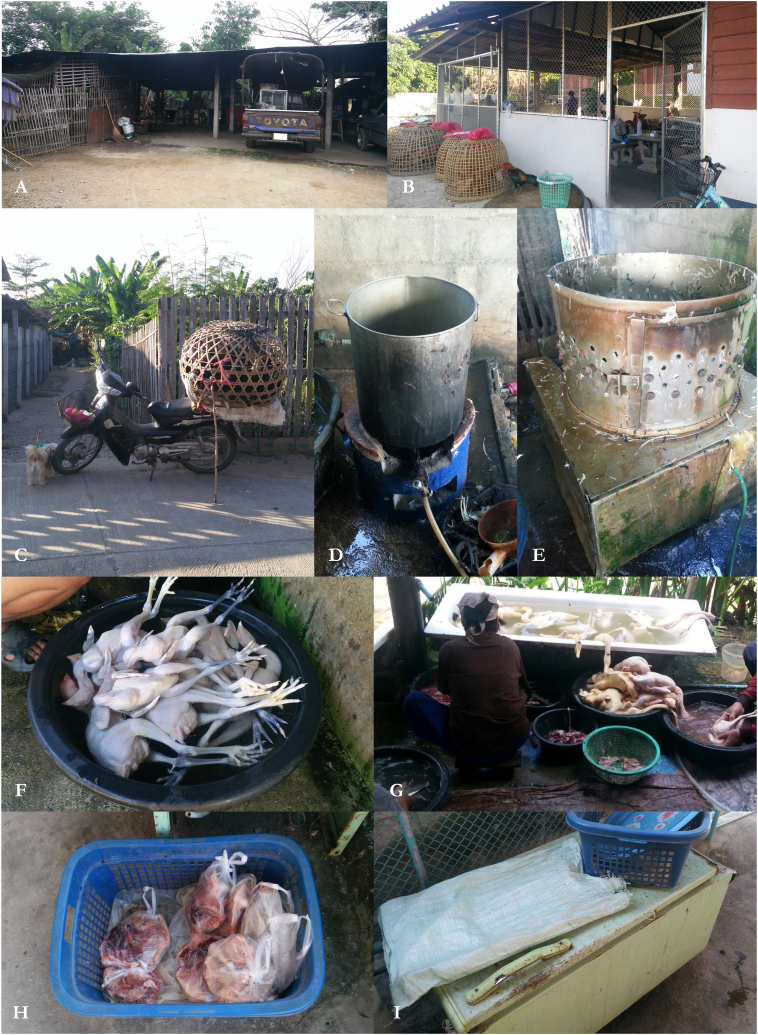
Photos of (**A**, **B**) backyard slaughterhouses; (**C**) chicken transportation from smallholders to a backyard plant; (**D**) scalding tank; (**E**) defeathering machine; (**F**) carcass soaking; (**G**) carcass evisceration process; (**H**) carcass packed with a plastic bag; (**I**) a modified, unused refrigerator filled with ice used for the storage of carcasses prior to distribution to the local market.

Backyard slaughterhouses, often using areas within the owners' homes for the slaughtering process (Fig. 3A and B), typically serve as a source of supplemental income for farmers due to the small daily production volume and the composition of chickens supplied for slaughtering. The majority of chickens supplied for slaughtering are either hens retired from layer chicken farms after completing their laying cycle or native chickens raised in backyards for additional household income. Suppliers for backyard slaughterhouses generally transport chickens from the rearing sites using motorcycles (Fig. 3C). In some cases, the slaughterhouse owners personally visit the farms to collect the chickens themselves. In contrast, the purpose of the commercial plants is to produce poultry meat products in large quantities for distribution in the province's urban areas and the northern region. The chickens are sourced directly from commercial broiler farms or contract farms. As the stated objective of the commercial plants, the basic concept of good manufacturing practices (GMPs) has been systematically adopted, such as biosecurity and worker personal hygiene, which can lead the slaughterhouses to pursue accreditation by government authorities and promote their commercialization to sell meat products at a higher price to higher markets, such as supermarkets, company's retail shops, and hotels. The number of estimated total backyard slaughterhouses in the study area may be underestimated due to the fact that the database for backyard slaughterhouses is out of date because there is no law or regulation requiring owners to register with the system. Some backyard plants are operating illegally because their proprietors used their homes or private property without registering with local authorities.

The slaughtering process and apparatus utilized (Fig. 3D and E) depend on the proprietors' knowledge and experience. As shown in Fig. 2, one of the most distinguishing practices between backyard and commercial plants is the killing method, where commercial plants have a standard to follow by using electrical stunning and bleeding, whereas backyard plants rely on the inhumane (no stunning) slaughter practices based on plant owner's expertise, e.g. neck hanging, throat cutting, and cervical dislocation without stunning. At the scalding and defeathering steps of backyard plants, half of the plants required employees to manually operate the steps, while the other half used a machine for defeathering. As the operation is primarily for generating additional income for the household, adapted equipment was utilized to reduce production costs, such as the use of a basin as a large container to soak the carcasses for the purposes of removing bloodstain and dirt as well as reducing carcass temperature (Fig. 3F and G). In the majority of cases, each chicken carcass is first packed in a plastic bag (Fig. 3H) and then placed in an unused refrigerator filled with ice (Fig. 3I), which has been modified to preserve the carcasses prior to sale.

For commercial plants, the majority of the processing steps were identical, with the exception of the temperatures observed at each step, and the washing and chilling steps. The CS1 was equipped with an automatic washer that washed the carcasses inside and outside after evisceration, whereas the CS2 combined manual and automated procedures. At the immersion chilling step, the CS1 utilized the overflow system to reduce carcass temperature and then cleaned the equipment at the end of each day's operations. The CS2 employed a static system (no water flow) in which water and ice were replaced at the end of each shift.

### 3.2 Sample profiles

**Fig. 4:**
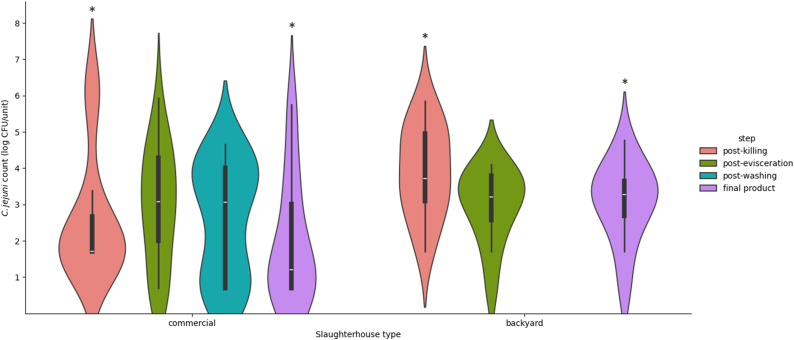
Violin plots showing probability density of the counts. Wider sections of the violin plot represent a higher probability that members of the samples will take on the given value; the skinnier sections represent a lower probability. The white dot represents the median, the thick black bar in the center represents the interquartile range, the thin black line represents the rest of the distribution, except for points that are determined to be outliers. The asterisks (*) above the violin plots indicate significant differences in counts (p-value <0.05) at that step between the commercial and backyard slaughterhouses.

Table 1 displays counts of *C. jejuni* contamination by type of slaughterhouse and by slaughtering step using mean, standard deviation (SD), 2.5th percentile, and 97.5th percentile. The count data exhibited a minor right skewness (skewness = 0.255), signifying that there were slightly more high-count values than low-count values. In commercial plants, the average *C. jejuni* count was highest at the post-evisceration step, whereas in backyard plants, it was highest at the post-killing step. In summary, the samples collected from commercial slaughterhouses exhibited a greater range of counts than those collected from backyard plants, as evidenced by the violin-shaped plots (Fig. 4). In addition, samples from commercial plants tended to have lower counts at each step than backyard plants, as evidenced by the lower medians and modes.

**Table 1:** Counts of *C. jejuni* contamination in chicken carcass a.

Type	Step	Mean	SD	2.5th percentile	97.5th percentile
Commercial	Post-killing	2.61	1.68	1.70	6.17
	Post-evisceration	2.95	1.60	0.70	5.42
	Post-washing	2.61	1.56	0.70	4.58
	Final product	2.00	1.70	0.70	5.62
Backyard	Post-killing	3.89	1.25	1.89	5.66
	Post-evisceration	2.98	1.01	0.98	4.04
	Final product	3.08	1.10	0.98	4.56

a Log CFU/g for cecal samples; log CFU/ml for carcass rinse samples. Final carcass products from commercial plants were carcasses that passed a chilling step. SD: standard deviation.

The Mann-Whitney *U* test showed a significant difference between the counts of samples collected at both the post-killing step (p-value = 0.01) and the final step (p-value = 0.04). Nevertheless, between commercial and backyard plants, the comparison of post-evisceration counts revealed no significant difference (p-value = 0.96) according to the independent *t*-test, and the comparison of total rinse sample counts revealed no significant difference (p-value = 0.20) according to the Mann-Whitney *U* test. As shown by these statistical tests, the analyses are intended to provide contamination profiles of the samples based on the type of slaughterhouse. Fig. 4 illustrates the distribution and probability density of *C. jejuni* counts at different steps of the process in both commercial and backyard slaughterhouses, and highlights the steps at which significant differences in bacterial counts were observed. Due to a lack of tracked samples, this investigation did not compare steps within the same type of slaughterhouse. Due to the aforementioned limitation, this does not provide conclusive evidence of contamination increment or reduction caused by a step within plants.

### 3.3 Cluster profiles

The k-modes clustering algorithm is utilized to classify data points into various clusters. As this type of machine learning is unsupervised and does not require a training process, it provides data insights by grouping data elements according to their categorical attributes. Because of the nature of the data acquired for this study, the majority of variables of interest are categorical. The implementation of k-modes clustering rather than k-means is therefore considered. Unlike k-means, which clusters continuous data using distance measures of the data points, k-modes uses dissimilarities between the data points. The closer together the data points are, the smaller the dissimilarities. Each cluster is characterized by its mode, which is the cluster's most frequent categorical value. For the ‘count’ of *C. jejuni* and ‘temperature’ at the collection step which are continuous data, four and six edges of the bins are the most appropriate numbers that can split data points into three categories for ‘count’ and five categories for ‘temperature’, respectively in order to transform continuous data into categorical. The categories for the ‘count’ variable in the cecal samples dataset would be defined as [1.7, 3.21), [3.21, 4.72), and [4.72, 6.23] for ‘Low’, ‘Medium’, and ‘High’ counts, respectively. The categories for the ‘count’ variable in the carcass rinse samples dataset would be defined as [0.7, 2.0), [2.0, 3.54), and [3.54, 6] for ‘Low’, ‘Medium’, and ‘High’ counts, respectively and the categories for the ‘temperature’ variable would be defined as [3.7, 5.1), [5.1, 22), [22, 26.8), [26.8, 42), and [42, 50.1] for ‘Very Low’, ‘Low’, ‘Medium’, ‘High’, and ‘Very High’ temperatures, respectively. The square brackets [] indicate that the endpoint is included in the set, while the parentheses (.) indicate that the endpoint is excluded from the set.

This study's data were divided into two datasets based on the sample types collected: cecal samples and carcass rinse samples. The elbow method was used to find the optimal number of clusters. As a result, we found that neither the cecal samples dataset nor the carcass rinse samples dataset had a distinct point of elbow. Thus, the number of clusters was set to three (K = 3) with the intention that the algorithm will cluster the data points based on counts (i.e. ‘Low,’ ‘Medium,’ and ‘High’) so that we could identify the patterns of practices that contributed to the different levels of contamination. As k-modes clustering is a type of unsupervised algorithm, we could not define its purpose explicitly. Therefore, if the clustering results did not meet our study objectives, we investigated and discussed other insights from the results.

**Fig. 5:**
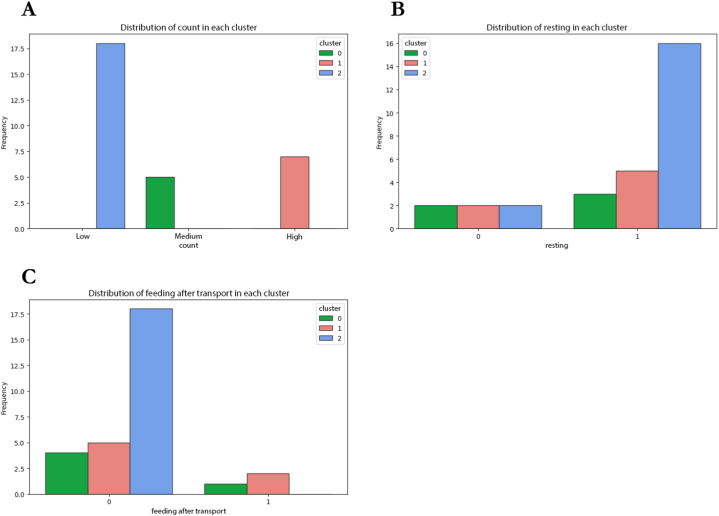
Distribution of various features within each identified cluster of cecal samples dataset. The bar plots show the frequency of the categorical values of the different features for each cluster, indicated by distinct colors. Each subplot corresponds to a different feature, e.g., (**A**) count category, (**B**) chicken resting, and (**C**) feeding practice after transportation, and the x-axis represents the categories of that feature (0 = No, 1 = Yes), while the y-axis shows the frequency of instances within each category. The distinct color-coded bars within each category represent the proportion of instances within each cluster. (For interpretation of the references to color in this figure legend, the reader is referred to the Web version of this article.)

**Fig. 6:**
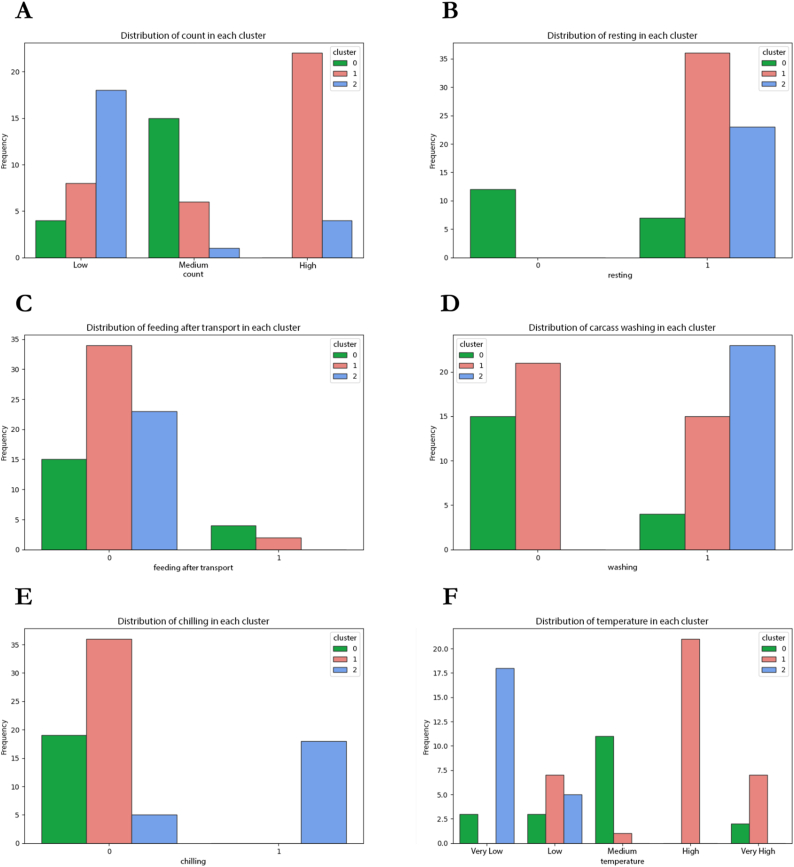
Distribution of various features within each identified cluster of carcass rinse samples dataset. The bar plots show the frequency of the categorical values of the different features for each cluster, indicated by distinct colors. Each subplot corresponds to a different feature, e.g., (**A**) count category, (**B**) chicken resting, (**C**) feeding practice after transportation, (**D**) carcass washing, (**E**) carcass chilling, and (**F**) temperature category at the step of sample collection, and the x-axis represents the categories of that feature (0 = No, 1 = Yes), while the y-axis shows the frequency of instances within each category. The distinct color-coded bars within each category represent the proportion of instances within each cluster. (For interpretation of the references to color in this figure legend, the reader is referred to the Web version of this article.)

Cluster profiles of cecal samples and carcass rinse samples were illustrated with bar plots in Fig. 5, Fig. 6, respectively. For cecal samples clustering, the groups were defined with clusters of Low, Medium, and High *C. jejuni* counts (Fig. 5A). From cluster distribution provided in Table 2, it could be implied that samples with low contamination of *C. jejuni* were from the commercial slaughterhouse (77.8 % of all samples in the cluster) while in the medium-count category, most of the samples were from backyard plants (80 %). However, based on count categories, types of slaughterhouses could not be comparable in terms of effectiveness of practices before a killing step, since there were close numbers of samples from both types of slaughterhouses in the high-count category. Additionally, practices of chicken resting before killing and feeding the chickens after transportation from farms to a slaughterhouse were not obviously identified between those three clusters, since the modes of those variables pointed to the same categories of chicken resting before killing (Fig. 5B) and fasting (no feed) of chickens after transportation to the slaughterhouse (Fig. 5C). Furthermore, for the variable ‘feeding’, only one backyard plant performed the practice of feeding the chickens before killing (Fig. 2). The proportions of chickens fed against those not fed were 10 % and 90 %, respectively. There were insufficient samples in this group to detect variations in feeding patterns or any insights from clustering. When dividing the counts into two categories, Low and High, and using K values of 2, no additional insight into the observed practices was acquired because no obvious resting or feeding behaviors were observed. Similar results were obtained when attempting to divide the counts into five categories (‘Very Low,’ ‘Low,’ ‘Medium,’ ‘High,’ and ‘Very High’) and employing K values of 5 (data not shown). In addition, fewer variables were considered for these samples, as this was the very first step of the slaughtering process; consequently, only limited clustering information was obtained.

**Table 2:** Cluster profiles of cecal samples dataset based on count category a.

Count category	Low	Medium	High
Number of samples in cluster	18	5	7
Count range	[1.7, 3.21) a	[3.21, 4.72)	[4.72, 6.23]
Cluster ID b	2	0	1
*Type of slaughterhouse* Backyard Commercial	4 (22.2 %) **14 (77.8 %)**^3^	**4 (80 %)** 1 (20 %)	**4 (57.1 %)** 3 (42.9 %)
*Chicken resting* No rest Rest	2 (11.1 %) **16 (88.9 %)**	2 (40 %) **3 (60 %)**	2 (28.6 %) **5 (71.4 %)**
*Chicken feeding* No feed Feed	**18 (100 %)** 0	**4 (80 %)** 1 (20 %)	**5 (71.4 %)** 2 (28.6 %)

a The square brackets [] indicate that the endpoint is included in the set, while the parentheses (.) indicate that the endpoint is excluded from the set.

b Cluster ID is referred to bar plots in Fig. 5.^3^ a description of the clusters of each feature, bold indicates the most common value.

For clustering carcass rinse samples, samples were classified into three clusters based on the most frequent value or mode (Fig. 6). Depending on the count categories, we discovered that the clusters can be divided into three categories (Fig. 6A). For the low-count cluster, all samples were collected from commercial plants, the majority of which (78.3 %) were collected post-chilling and represented as the final product. As shown in Table 3, the samples in this cluster were collected at very low and low temperatures. In the medium-count cluster, the majority of samples (84.2 %) were from backyard plants, and the modes indicated that neither the poultry nor their carcasses were rested, fed, washed, or chilled (Fig. 6B–E). The samples in the group were collected at a range of temperatures, with the majority collected at moderate temperatures (22–26.8 °C). Intriguingly, in the high-count cluster, the majority of samples (77.8 %) came from commercial plants, and the majority were collected after evisceration (55.6 %) and washing (33.3 %), respectively. The modes of practice included no pre-slaughter resting of poultry (100 %), no feeding (94.4 %), and no washing of carcasses (58.3 %). In addition, the majority of these samples were collected at elevated temperatures (26.8–42 °C) (Fig. 6F).

**Table 3:** Cluster profiles of carcass rinse samples dataset based on count category.

Count category	Low	Medium	High
Number of samples in cluster	23	19	36
Count range	[0.7, 2) a	[2, 3.54)	[3.54, 5.93]
Cluster ID b	2	0	1
*Type of slaughterhouse* Backyard Commercial	0 **23 (100 %)**^3^	**16 (84.2 %)** 3 (15.8 %)	8 (22.2 %) **28 (77.8 %)**
*Step* Post-evisceration Post-washing Final product	– 5 (21.7 %) **18 (78.3 %)**	**10 (52.6 %)** 1 (5.3 %) 8 (42.1 %)	**20 (55.6 %)** 12 (33.3 %) 4 (11.1 %)
*Chicken resting* No rest Rest	0 **23 (100 %)**	**12 (63.2 %)** 7 (36.8 %)	0 **36 (100 %)**
*Chicken feeding* No feed Feed	**23 (100 %)** 0	**15 (78.9 %)** 4 (21.1 %)	**34 (94.4 %)** 2 (5.6 %)
*Carcass washing* No wash Wash	0 **23 (100 %)**	**15 (78.9 %)** 4 (21.1 %)	**21 (58.3 %)** 15 (41.7 %)
*Carcass chilling* No chill Chill	5 (21.7 %) **18 (78.3 %)**	**19 (100 %)** 0	**36 (100 %)** 0
*Temperature before collection* Very Low [3.7, 5.1) Low [5.1, 22) Medium [22, 26.8) High [26.8, 42) Very High [42, 50.1]	**18 (78.3 %)** 5 (21.7 %) 0 0 0	3 (15.8 %) 3 (15.8 %) **11/(57.9 %)** 0 2 (10.5 %)	0 7 (19.4 %) 1 (2.8 %) **21 (58.3 %)** 7 (19.4 %)

a The square brackets [] indicate that the endpoint is included in the set, while the parentheses (.) indicate that the endpoint is excluded from the set.

b Cluster ID is referred to bar plots in Fig. 6.^3^ a description of the clusters of each feature, bold indicates the most common value.

## 4 Discussion

The purpose of this investigation was to improve understanding of the differences between backyard and commercial slaughtering practices in food hygiene. Due to the implementation of good manufacturing practices (GMPs), we hypothesized that commercial slaughterhouses performed better than backyard slaughterhouses. However, because of the limitations of slaughterhouse access, particularly for backyard plants, the quantity of samples gathered was limited, preventing us from directly comparing the operations of the two types of slaughterhouses. In addition, in order to analyze the risk of *C. jejuni* contamination in chicken meat as part of the project, the carcass rinsing technique was used to quantify the contamination of whole carcasses. Therefore, sample tracking was not utilized in the study. Following the collection of samples and data, we utilized the benefits of machine learning to gain insight.

As *C. jejuni* bacteria grow and multiply in the chicken's lower gastrointestinal tract [27,28], chicken carcasses are contaminated with cecal content during critical slaughtering steps, such as defeathering and evisceration [[29], [30], [31], [32]], and reach consumers. Thus, the primary level of production, i.e. farms, is the primary source of contamination, and minimizing the number of the bacteria in carcasses prior to processing and distributing should ideally be targeted at this level [5,6,33]. However, controlling contamination at farms is difficult due to the horizontal transmission of the bacteria in broiler flocks from vectors during rearing [6,31], including contamination of feed and water and cross-contamination during chicken transportation to the slaughterhouse [34]. *C. jejuni* contamination in chicken meat is of less concern than other foodborne pathogens, such as *Salmonella* spp., because the level of contamination is not rigorously regulated for domestic consumption in the country. However, due to the potentially detrimental public health effects of campylobacteriosis [1], slaughtering interventions are necessary to reduce the level of *C. jejuni* contamination in carcasses prior to distribution for human consumption.

Due to the lack of step-by-step traceability of the samples used in this study, it is challenging to determine which slaughterhouse type is more effective in terms of best practices. Individual carcasses were collected at each step. We utilized the benefits of carcass rinsing to provide a more comprehensive assessment of the bacterial contamination, as the sample collected and the technique employed represent the entire carcass contamination, as opposed to using neck skin samples. There were significant differences between counts of *C. jejuni* in cecal samples collected from backyard and commercial plants, with backyard plants having approximately 1 log CFU/g more *C. jejuni* contamination. The mean counts of *C. jejuni* in ceca observed in this study in both commercial and backyard plants were lower than those reported by Giombelli and Gloria (2014) and Stern et al. (1995), who reported mean counts of 5.43 log CFU/g and 6.15 log CFU/g, respectively [13,35]. This could be due to the randomness of samples or differences in factors that contributed to the lower number of *C. jejuni*, such as the season of sample collection, age at slaughter, feed withdrawal time, transportation time, or other farm management factors, including biosecurity, age of houses, downtime, and flock size [36,37]. In this study, the cecal samples were representative of *C. jejuni* colonization in poultry from farms and other sources of rearing. Backyard or free-range poultry are exposed to an outdoor environment with less control over *Campylobacter* contamination than chickens raised in a conventional system with biosecurity measures and a controlled environment [38]. As chickens supplied to backyard slaughterhouses come from spent laying hens or hens processed for meat after egg production declines, as well as native chickens from households, the significantly longer period of rearing in several months, compared to 6–7 weeks for the conventional system [39], makes them longer exposed to multiple sources of contamination, namely other farms and wild animals as well as soil [11,33,38], and reinfection with *C. jejuni*, resulting in elevated levels of the pathogen. Counts of *C. jejuni* at the final step revealed that the average counts of *C. jejuni* in samples collected from commercial plants were approximately 1 log CFU/g lower than those from backyard plants. However, we did not find any statistically significant differences in the counts of samples collected at subsequent steps after killing, and sample tracking was not implemented in this study; therefore, additional analysis was conducted to gain more insight into slaughtering practices and *C. jejuni* contamination.

This is the first study to our knowledge that employs k-modes clustering to analyze the relationship between various slaughtering practices and the level of *C. jejuni* contamination in chicken carcasses, providing novel insights into the significance of each slaughtering step. Due to the categorical nature of the majority of the variables of interest in our dataset, we opted for k-modes clustering. K-modes is optimal for categorical data because it measures dissimilarity between data points rather than distance, making it more applicable for this data type [40]. In contrast to k-means, which is widely used for clustering continuous data, k-modes does not require the calculation of the mean of variables. The k-means method calculates the centroid of clusters by calculating the mean of all elements within a cluster [41], which is neither applicable nor meaningful when working with categorical variables. K-modes, on the other hand, designate a mode for each cluster that corresponds to the most prevalent category of variables within that cluster, making it more appropriate for categorical data. By choosing k-modes over k-means, we aimed to obtain more precise and informative clusters, thereby obtaining a deeper understanding of our data and its patterns.

The clustering results of cecal samples are consistent with the descriptive analysis, indicating that samples from commercial plants were less contaminated than those from the backyard, as the majority of samples in the low-count category were from commercial plants. As the rest of the variables (resting and feeding) were not comparable because the modes are the same for the three clusters, differences in counts in cecal samples may refer to the management of chicken rearing as previously mentioned rather than the practices of resting and feeding/fasting the chickens prior to slaughter as these take a short amount of time and may not have a noticeable effect on contamination increment or reduction. This is also evident in the clustering of carcass rinse samples, where the practices of poultry feeding and resting cannot be distinguished between the low-, medium-, and high-count groups.

Based on the cluster profiles of carcass rinse samples, it is evident that all of the samples in the low-count group passed the washing process and the majority were chilled in the final production step. As evidenced by other studies, it can be inferred that chilling is the crucial step for reducing and maintaining the lower level of contamination [[42], [43], [44], [45], [46]]. High loads of *C. jejuni* at the entering step of slaughter operations or high loads of *C. jejuni* colonization from farms have an effect on the number of bacteria at subsequent steps. Reich et al. (2008) and Hue et al. (2010) found a positive correlation between the number of *C. jejuni* in the ceca and the number of the pathogen found on processed carcasses [47,48]. In addition, it is hypothesized that a higher initial level of *C. jejuni* contamination results in less reduction after processing and, thus, a higher level of the bacteria in the final products [43]. Consequently, the final step of slaughtering prior to the distribution of meat products is crucial in determining the level of contamination and poses a threat to consumer safety. As commercial facilities employ immersion chilling, if the step of reducing contamination exists as the very last stage of processing, it can help reduce the number of *C. jejuni* contaminations prior to distribution to retailers and consumers, respectively. Note that for the ‘chill’ variable used for clustering, all of the samples collected as final products from the backyard plants are coded as zero because they were collected immediately after packaging. Therefore, it is recommended that practices for preserving carcasses at low temperatures prior to sale be implemented in backyard slaughterhouses and that further research be conducted on the efficiency of low temperature carcass preservation as well as the tracking of samples from the initial to final critical steps to determine whether carcass preservation techniques reduce *C. jejuni* contamination. Immersion chilling, which is utilized in commercial plants, can also assist in reducing *C. jejuni* loads because it encourages the washing of carcasses with adequate chlorine concentrations and reduces carcass temperature to an unsuitable level for *C. jejuni* growth [44]. Further investigation is warranted to assess the impact of various chilling parameters, including chilling rate, duration, chlorine concentration, and carcass weight, on the reduction of *C. jejuni*. These aspects are comprehensively reviewed in the work by James et al. (2006) [49]. Understanding these variables is pivotal for the refinement of chilling protocols, which could significantly contribute to enhancing the microbial safety of poultry production.

Notably, nearly half of the samples in the cluster with the highest count also passed the washing process, but not the chilling step. As previously reported in the study by Bashor et al. (2004), a washing step has a minor effect on the reduction of *C. jejuni* numbers compared to the chilling step, which could significantly reduce the number of *C. jejuni* [50]. This may be due to the washing step, which dilutes the concentration of *C. jejuni*. However, cross-contamination of the bacteria by machinery or personnel may be occurring during the process [51]. Additionally, carcass washing at commercial slaughterhouses in this study was performed with chlorinated treated water at normal concentrations for human consumption (0.5–1 ppm), which may have been less effective in reducing *C. jejuni* than the use of chlorinated water with higher concentrations [52] and the application of processing aids such as trisodium phosphate (TSP) and acidified sodium chlorite (ASC) spraying [47]. Furthermore, *C. jejuni* prefers elevated temperatures, as the bacteria requires a growth temperature range of 30–47 °C [53]. This is consistent with the clustering results indicating that the majority of samples in the cluster with high counts were collected at high temperatures (26.8–42 °C).

Due to the predominantly categorical nature of our data set, we utilized the k-modes clustering technique in our study. This method, in contrast to k-means, does not rely on the calculation of means; instead, it concentrates on the modes of categories, thus meeting our specific requirements. Despite the fact that the efficacy of the k-modes algorithm is not intrinsically dependent on sample size, the accuracy, stability, and interpretability of our clustering results can be affected by the number of samples used. Smaller sample sizes may not capture the complete complexity and underlying structure of the data, potentially compromising the robustness of the findings.

However, there are some limitations to our investigation that must be acknowledged. The limited number of participating slaughterhouses resulted in a small sample size, which is the primary limitation. As a result, it became impossible to subcategorize backyard slaughterhouses based on slightly differences of practices at some steps. In addition, the types of samples collected at the initial step differed from those collected at later steps, as our goal was to determine the initial contamination of *C. jejuni* from rearing prior to implementing other crucial operational steps. A step prior to evisceration, specifically pre- or post-scalding and defeathering, should be considered to establish a baseline contamination level of the carcass for a more comprehensive understanding of contamination levels. This would enable for a more accurate comparison of the effects of different slaughterhouse intervention methods. For future studies, it is suggested that a more detailed system for tracking samples throughout the processing chain would provide more granular insight into variations in contamination levels. Identifying specific actions with substantial effects on *C. jejuni* contamination could pave the way for targeted interventions to improve sanitation and lower pathogen levels.

In consideration of future research, we identified patterns and classifications that could serve as the basis for future studies. There may be additional factors influencing the level of *C. jejuni* contamination in chicken carcasses that were not addressed by our research, despite the fact that our study has yielded significant insights. A deeper examination of the characteristics defining these clusters and their interactions with various slaughtering practices would be beneficial. In addition, more extensive research could isolate and evaluate the impact of specific practices or conditions that contribute to variations in *C. jejuni* counts. Furthermore, future research would benefit greatly from implementing other machine learning techniques, such as supervised learning algorithms, to predict contamination levels based on the variables identified in this study.

Additionally, as suggested by our clustering results, future research could concentrate on effective methods for implementing improved practices in both backyard and commercial slaughterhouses. A trial evaluating interventions intended at reducing *C. jejuni* counts, particularly at stages where high contamination levels were detected, could be beneficial. Lastly, while our study was predominately based on categorical attributes, future research could consider continuous attributes, where applicable, and use other clustering methods such as k-means or hierarchical clustering for a more comprehensive view. This could uncover new data relationships and provide additional insights.

## 5 Conclusion

This study revealed a significant difference in *C. jejuni* contamination between commercial and backyard poultry slaughterhouses, emphasizing the critical role of enhanced slaughtering practices in controlling bacterial contamination. The findings demonstrate the significance of chilling in reducing *C. jejuni* levels and the need for improved practices, particularly in backyard operations. Backyard operations could benefit from adopting practical measures seen in commercial slaughterhouses, such as the segregation of equipment at each critical step. Employing separate basins for soaking carcasses before and after evisceration, coupled with regular equipment cleaning, may potentially mitigate the risk of cross-contamination. Additionally, given the clustering results that indicate a decrease in *C. jejuni* levels, integrating ice into the post-evisceration soaking process is advisable. This approach not only lowers the carcass temperature but also helps to reduce the concentration of *C. jejuni*, thereby enhancing the meat's safety before it reaches consumers. Utilizing k-modes clustering provided an innovative method for interpreting complex data in the context of food safety. Additional research is required to refine strategies and interventions for reducing *C. jejuni* contamination and to leverage machine learning to predict contamination levels based on operational practices. These findings should motivate efforts to improve poultry slaughterhouse management and reduce foodborne illnesses.

## Funding

This research was funded by the Excellent Center of Veterinary Public Health, 10.13039/501100002842Chiang Mai University (R000016652). The funder had no role in study design, data collection and analysis, decision to publish, or preparation of the manuscript.

## Ethical approval statement

Review and/or approval by an ethics committee was not needed for this study because it focused solely on the collection of chicken carcass samples for bacterial enumeration and the gathering of observational data at slaughterhouses, without any direct interaction or manipulation of live animals. The samples were obtained following the standard slaughter process, and data were collected through observation of slaughterhouse operations, not involving human subjects in a manner requiring ethical review. Prior consent for carcass sampling and photography was obtained verbally from slaughterhouse owners. Care was taken to ensure that photographs did not compromise personal identification, further supporting the determination that formal ethics approval was not required.

## Data availability statement

Data will be made available on request.

## CRediT authorship contribution statement

**Chalita Jainonthee:** Writing – review & editing, Writing – original draft, Visualization, Software, Investigation, Formal analysis, Data curation. **Warangkhana Chaisowwong:** Writing – review & editing, Supervision, Project administration, Methodology, Funding acquisition, Conceptualization. **Phakamas Ngamsanga:** Writing – review & editing, Investigation. **Tongkorn Meeyam:** Writing – review & editing, Supervision, Methodology, Conceptualization. **Fernando Sampedro:** Writing – review & editing, Validation, Supervision, Conceptualization. **Scott J. Wells:** Writing – review & editing, Validation, Supervision. **Duangporn Pichpol:** Writing – review & editing, Validation, Supervision, Resources, Project administration, Methodology, Funding acquisition, Formal analysis, Conceptualization.

## Declaration of competing interest

The authors declare the following financial interests/personal relationships which may be considered as potential competing interests:Duangporn Pichpol reports article publishing charges was provided by Chiang Mai University. If there are other authors, they declare that they have no known competing financial interests or personal relationships that could have appeared to influence the work reported in this paper.
